# CRISPR/Cas9 Allows Efficient and Complete Knock-In of a Destabilization Domain-Tagged Essential Protein in a Human Cell Line, Allowing Rapid Knockdown of Protein Function

**DOI:** 10.1371/journal.pone.0095101

**Published:** 2014-04-17

**Authors:** Arnold Park, Sohui T. Won, Mickey Pentecost, Wojciech Bartkowski, Benhur Lee

**Affiliations:** 1 Department of Microbiology, Immunology and Molecular Genetics, University of California Los Angeles, Los Angeles, California, United States of America; 2 Department of Microbiology, Icahn School of Medicine at Mount Sinai, New York, New York, United States of America; Temple University, United States of America

## Abstract

Although modulation of protein levels is an important tool for study of protein function, it is difficult or impossible to knockdown or knockout genes that are critical for cell growth or viability. For such genes, a conditional knockdown approach would be valuable. The FKBP protein-based destabilization domain (DD)-tagging approach, which confers instability to the tagged protein in the absence of the compound Shield-1, has been shown to provide rapid control of protein levels determined by Shield-1 concentration. Although a strategy to knock-in DD-tagged protein at the endogenous loci has been employed in certain parasite studies, partly due to the relative ease of knock-in as a result of their mostly haploid lifecycles, this strategy has not been demonstrated in diploid or hyperploid mammalian cells due to the relative difficulty of achieving complete knock-in in all alleles.

The recent advent of CRISPR/Cas9 homing endonuclease-mediated targeted genome cleavage has been shown to allow highly efficient homologous recombination at the targeted locus. We therefore assessed the feasibility of using CRISPR/Cas9 to achieve complete knock-in to DD-tag the essential gene Treacher Collins-Franceschetti syndrome 1 (TCOF1) in human 293T cells. Using a double antibiotic selection strategy to select clones with at least two knock-in alleles, we obtained numerous complete knock-in clones within three weeks of initial transfection. DD-TCOF1 expression in the knock-in cells was Shield-1 concentration-dependent, and removal of Shield-1 resulted in destabilization of DD-TCOF1 over the course of hours. We further confirmed that the tagged TCOF1 retained the nucleolar localization of the wild-type untagged protein, and that destabilization of DD-TCOF1 resulted in impaired cell growth, as expected for a gene implicated in ribosome biogenesis. CRISPR/Cas9-mediated homologous recombination to completely knock-in a DD tag likely represents a generalizable and efficient strategy to achieve rapid modulation of protein levels in mammalian cells.

## Introduction

To determine the functional contribution of a cellular gene to a biological process, a mRNA knockdown or genomic knockout approach is often used. When it comes to genes that are critical for cell viability and growth, however, a knockdown or knockout approach may lead to sickly or nonviable cells that make appropriate comparison to control cells difficult or impossible. Further, RNA knockdown can take days to result in substantial reduction in protein levels, especially if the protein has a long *in vivo* half-life.

To address these concerns and achieve rapid knockdown of an essential gene in mammalian cells, we combined two recent approaches: i) the use of a destabilizing FKBP protein-based 12 kDa tag to confer chemical compound-dependent regulation of protein levels [1], and ii) the CRISPR/Cas9 bacterial homing endonuclease system that allows efficient homologous recombination at endogenous loci [2], [3]. The resultant destabilization domain (DD)-tagged protein, when stabilized, allows cell growth and viability, whereas removal of the stabilizing compound results in rapid knockdown at the protein level. Although a knock-in DD-tagging strategy has been employed in parasite models such as *Plasmodium falciparum* and *Toxoplasma gondii* [4], [5] (also see [6], [7] for recent examples), this has been due to the relative ease of modification of the usually haploid genome; in diploid and hyperploid mammalian cell lines, however, the relative difficulty of complete knock-in in multiple alleles has been a barrier to use of this DD-tagging knock-in strategy. The demonstrated efficiency of CRISPR/Cas9 in stimulating targeted homologous recombination made it reasonable to attempt this knock-in strategy in a standard human cell line.

We chose to test the Cas9-mediated DD-tag knock-in strategy on Treacher Collins-Franceschetti syndrome 1 (TCOF1, also known as treacle), an essential gene encoding a nucleolar protein that is important for ribosome biogenesis, neural crest cell development and neurogenesis [8]–[12]. Haploinsufficiency of TCOF1 in humans is associated with Treacher Collins syndrome, characterized by impaired craniofacial development as well as ear defects leading to deafness [13]–[16], and the absence of TCOF1-null mice or humans suggests that knockout is embryonic lethal. Haploinsufficiency in mice can result in neonatal death or survival, depending on the genetic background [8], [17]. Further, recent genome-wide knockout screens using CRISPR/Cas9 in various human cell lines confirmed that TCOF1 is important for cell viability in all cell types tested [18], [19]. Within three weeks of initially transfecting the appropriate TCOF1-specific Cas9 and donor plasmids, we succeeded in expanding numerous single cell clones of human 293T cells with complete knock-in of DD-tagged TCOF1, despite the presence of at least three alleles of TCOF1 in these transformed cells. The compound Shield-1 stabilized DD-TCOF1 in a dose-dependent manner, and the removal of Shield-1 resulted in a several-fold decrease in DD-TCOF1 expression. Consistent with the known role of TCOF1 in ribosome biogenesis, destabilization of TCOF1 resulted in a significant decrease in cell growth.

To the best of our knowledge, this report represents the first demonstration that knock-in of DD-tagged protein in mammalian cells via CRISPR/Cas9 is an efficient strategy for rapid knockdown at the protein level.

## Materials and Methods

### Cell line and plasmids

**Figure 1:**
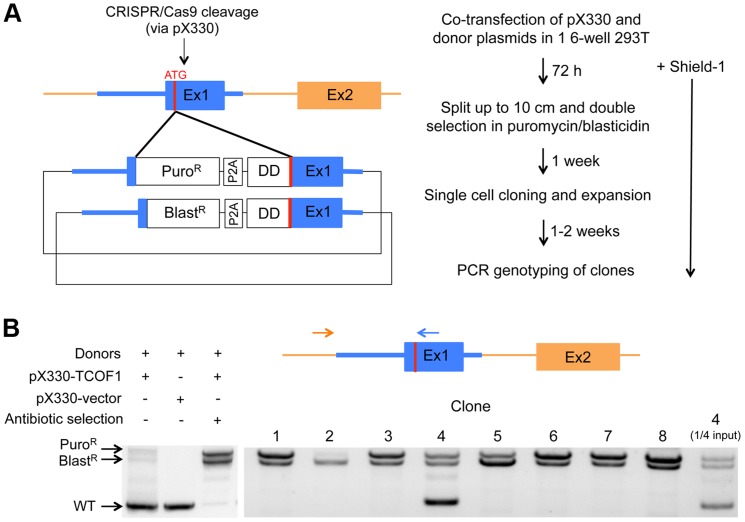
CRISPR/Cas9 allows efficient and complete endogenous knock-in of DD-tagged TCOF1. (**A**) The scheme for CRISPR/Cas9-mediated homologous recombination to insert antibiotic resistance and a DD tag to the N-terminus of TCOF1 is shown. The genomic sequence used in the donor plasmid homology arms is shown in blue. (**B**) PCR genotyping to detect knock-in was performed with the primers indicated on the diagram, and reveals wild-type (960 bp), blasticidin resistance (Blast^R^) knock-in (1790 bp), and puromycin resistance (Puro^R^) knock-in (1990 bp) bands. ¼ of the clone 4 PCR was also run to illustrate the lack of wild-type allele detection in the other clone PCRs.

293T cells were maintained in DMEM with 10% FCS and supplemented with penicillin/streptomycin. The pX330 CRISPR/Cas9 plasmid, designed by Cong *et al*. [2], was obtained from Addgene (plasmid 42230). For detailed discussion on CRISPR/Cas9 systems and applications, Drs. George Church and Feng Zhang among others maintain helpful resources and FAQs online as well, which can be found through the Addgene website (http://www.addgene.org/CRISPR/). The TCOF1-specific guide RNA sequence (Figure S1) was chosen from the curated database from Mali *et al.* [3] and inserted into the pX330 BbsI cloning site as described [20]. For the donor plasmids (Figure 1A and S2), the left and right homology arms were PCR-amplified using Velocity DNA Polymerase (Bioline) from 293T genomic DNA (isolated using the PureLink Genomic DNA Kit, Invitrogen). The destabilization domain was PCR-amplified from pTRE-Cycle1 (Clontech). The homology arms, antibiotic resistance ORF, and destabilization domain were joined together using standard overlapping PCR, then inserted between the BamHI and EcoRI sites of the PCR2.1 vector (Invitrogen) using In-Fusion (Clontech). 6XHis-tagged FKBP12 with the F36V mutation (on which the destabilization domain was based) in pET-15b (Novagen) was a kind gift of Dr. Thomas Wandless. FKBP-F36V was purified from BL21(DE3) *E. coli* as previously described [21] with minor modifications. Briefly, cells at OD_600_ 0.5 were induced with 2 mM IPTG at 37°C for 3 hours, then collected and lysed in sodium phosphate buffer (pH 8) containing 10 mM imidazole. 6XHis-tagged FKBP was purified on a 5 mL HisTrap HP (GE Healthcare) column via FPLC, with increasing imidazole concentration up to 250 mM. Pure fractions containing FKBP protein were pooled and dialyzed overnight into PBS.

### Transfection and selection

293T cells at 70% confluence in a 6-well were co-transfected with 1 µg each of pX330 (with TCOF1-specific guide sequence inserted), puromycin resistance donor plasmid, and blasticidin resistance donor plasmid, with 4.5 µL BioT transfection reagent (Bioland). At 24 hours post-transfection, media was changed with 100 nM Shield-1 (Clontech), and cells were subsequently always maintained in Shield-1. At 72 hours post-transfection, the 6-well was trypsinized and seeded into a 10 cm dish with 1 µg/mL puromycin and 3 µg/mL blasticidin. The media with antibiotics was changed every 3 days. Upon complete cell death in the controls (untransfected cells in single puromycin or blasticidin selection) after a week of selection, cells were trypsinized and plated in two 96-wells at a seeding density of 0.3 cells per well. Wells were monitored for single colony formation and expanded upon confluency.

### PCR genotyping of knock-in clones

Genomic DNA was isolated using the PureLink Genomic DNA Kit (Invitrogen) and genotyped using Velocity DNA Polymerase (Bioline) and the following primers: TCOF_screen-F: GTTTAGGGTTCCCAGGCAAT and TCOF_screen-R: CAGCAGATGGTGGTAGATC (positions in genomic sequence shown in Figure S1). For the PCR shown in Figure S3, the primers used were TCOF_screen-F and also TCOF_screen-R2: CCACATGAGTAGCCAGGATTAC.

### Western analysis of TCOF1 expression

**Figure 2:**
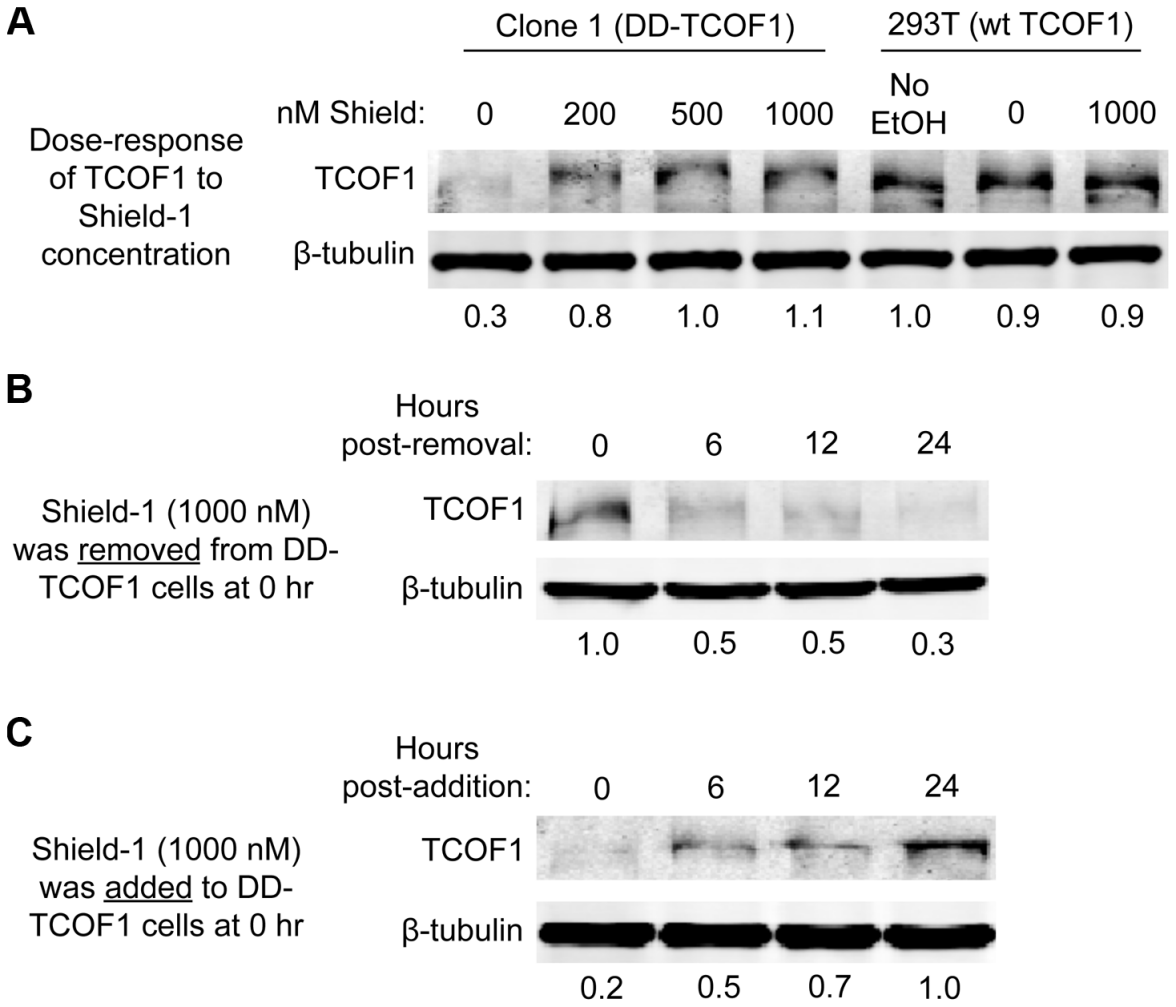
DD-TCOF1 expression is regulated by the concentration of Shield-1. (**A–C**) The Shield-1 concentration- and time-dependent expression of TCOF1 was examined as described in Materials and Methods. Although the EtOH vehicle was kept constant as described, a control without EtOH vehicle was also included for the parental wild-type 293Ts in panel A. As wild-type TCOF1 runs at about 220 kDa, the addition of the 12 kDa DD tag results in small upward shift for knock-in cells. Two-fold dilutions of high TCOF1-expressing sample were included on each gel to allow for standard curves to quantitate relative TCOF1 and β-tubulin. Relative TCOF1 values normalized to β-tubulin are shown below each panel. The data represents one of three experiments with similar results.

3×10^5^ knock-in or parental 293T cells per well were plated in 12-well in no Shield-1 (Figure 2A and C) or 1000 nM Shield-1 (Figure 2B). The following day, for addition of Shield-1, media was simply replaced with the appropriate concentration, with EtOH (the vehicle for Shield-1) remaining constant. For removal of Shield-1, media was replaced with conditioned media supplemented with 5 µM FKBP-F36V for 30 minutes, followed by continued incubation in media without Shield-1. At the indicated time points (24 hours for Figure 2A), cells were collected in cold PBS with 10 mM EDTA, pelleted, lysed in cold lysis buffer (0.1% NP-40, 100 mM Tris-HCl pH 7.5, 150 mM NaCl, 5% glycerol, 1 mM sodium orthovanadate, 1 mM EDTA, 1X protease inhibitor cocktail (Roche)), clarified at 13 k rpm for 5 minutes at 4°C, then stored at -20°C until use. A small portion of cells was reserved for cell counting. Lysates were boiled with Laemmli SDS sample buffer, and the equivalent of 4×10^4^ cells were run per sample on 8% Tris-glycine SDS-PAGE. Gels were transferred to PVDF (Immobilon-FL, Millipore) and blocked in Odyssey Blocking Buffer (LI-COR Biosciences) overnight at 4°C. Blots were then incubated in 1∶1000 rabbit anti-TCOF1 (11003-1-AP, Proteintech) or 1∶100,000 mouse anti-β-tubulin (T7816, Sigma) for 1 hour at room temperature, followed by 1∶10,000 of fluorescent secondary antibody (goat anti-rabbit IRDye 800CW or goat anti-mouse IRDye 680LT, LI-COR Biosciences). Fluorescence images were obtained on a LI-COR Odyssey imaging system.

### Immunofluorescence staining and data analysis

Wild-type or knock-in cells were plated in EtOH vehicle only or 1000 nM Shield-1 on coverslips that had been coated sequentially with poly-L-lysine and collagen. 24 hours post-plating, cells were fixed in 2% paraformaldehyde, washed 3 times with PBS, incubated in blocking buffer (0.5% saponin, 3% BSA in PBS), then incubated with 1∶200 rabbit anti-TCOF1 (11003-1-AP, Proteintech), 1∶500 mouse anti-nucleolin (39-6400, Sigma), and 1∶250 Alexa Fluor 647 Phalloidin (Invitrogen) in blocking buffer. After 3 washes in 0.5% saponin in PBS, samples were incubated with 1∶1000 secondary antibodies goat anti-rabbit Alexa Fluor 488 and goat anti-mouse Alex Fluor 594 (Invitrogen). After 3 final washes in 0.5% saponin in PBS, samples were incubated with DAPI to stain nuclei before mounting on slides. Confocal imaging was performed on a Leica SP5 confocal microscope, acquiring optical Z-stacks of 0.3 µm steps. Z-stacks were reconstructed and analyzed in three dimensions using Volocity 5.5 software (Perkin Elmer). Individual nuclei were localized and defined in three dimensions by DAPI staining. DNA-absent regions (such as nucleoli) were included in the defined nucleus, touching nuclei were separated, and fluorescence objects smaller than nuclei were excluded from analysis. Fluorescence voxel intensities in all channels were then measured within these computationally defined nuclei.

### Growth curves

2×10^4^ cells per well for wild-type 293T cells or knock-in cells were plated from master mix cell suspensions into either EtOH- or Shield-1-containing media in 6-well plates. Beginning 1 day post-plating and each day thereafter, cells were trypsinized and counted (in triplicate for each condition). The media was changed on day 3 for cells to be collected on subsequent days.

## Results

### CRISPR/Cas9 allows efficient and complete knock-in of destabilization domain-tagged TCOF1 at endogenous loci

The double-strand breaks mediated by CRISPR/Cas9 can stimulate homologous recombination in the presence of a DNA donor with appropriate homology arms. We first modified the pX330 Cas9-expressing plasmid [2] to specifically target the human genome near the start codon of TCOF1 (Figure 1A and S1). We then created donor plasmids that would result in knock-in of a DD tag linked to the N-terminus of TCOF1 via a 3XGGGGS flexible linker. To select for cells with knock-in at more than one allele, thus simplifying our screening process for clones with complete knock-in, we designed two donor plasmids with either puromycin or blasticidin resistance, followed by a P2A ribosomal skipping sequence and the DD tag and linker, all flanked by 800 bp homology arms (Figure 1A and S2). The P2A sequence appears to be the most efficient of the 2A skipping sequences in human cell lines [22], and we also included a GSG linker immediately preceding the P2A sequence (Figure S2), which has been shown to ensure essentially complete skipping [23], [24]. Upon knock-in, antibiotic resistance is driven off the endogenous promoter, and the DD-tagged protein is translated from the same mRNA transcript via the P2A sequence (Figure 1A). Double antibiotic selection should therefore select for cells with at least two modified alleles.

A clonal population of 293T cells was co-transfected with the TCOF1-specific pX330 Cas9 plasmid and the two donor plasmids (Figure 1A). At one day post-transfection, the media was changed to include 100 nM Shield-1 to stabilize DD-TCOF1, and Shield-1 was continually present in the media after this point through the entire knock-in process. At three days post-transfection, the cells were expanded and incubated with puromycin and blasticidin at predetermined minimal concentrations. After a week of antibiotic selection (control cells were all dead at this point in either single puromycin or blasticidin selection), cells were collected and single cell-cloned into 96-well dishes with no more subsequent antibiotic selection. Over the next 1-2 weeks, clones were expanded up to two 12-wells, from which one well was frozen for subsequent use and the other well was collected for genomic DNA isolation.

Next, we genotyped the clones by PCR to determine whether knock-in at the endogenous loci had occurred. To ensure specificity for the endogenous locus, while the reverse primer was specific for sequence within the right (3′) homology arm present in the donor plasmids, the forward primer was specific for genomic sequence beyond the left (5′) homology arm, and thus not present in the donor plasmids (Figure 1B). PCR of the wild-type locus results in a 960 bp band, whereas PCR of the knock-in allele results in 1990 and 1790 bp bands for puromycin or blasticidin resistance, respectively. We thus genotyped the eight fastest growing clones, and 7/8 resulted in complete knock-in (Figure 1B). The presence of wild-type and puromycin/blasticidin alleles in clone 4 indicates that there are at least three alleles of TCOF1 in these genetically unstable cells; this is also evident in the apparent predominance of either the puromycin or blasticidin allele in any given clone, indicating more of one or the other allele (note the difference between clones 5 and 6, for example). Although the smaller PCR fragment was useful to better distinguish the size difference between the puromycin/blasticidin alleles, we further confirmed recombination at the right homology arm by using primers specific for genomic sequence beyond both homology arms (Figure S3). For subsequent experiments, we used clone 1 as shown in Figure 1B.

As further evidence for the utility of Cas9 as well as antibiotic selection for this knock-in strategy, 293T cells transfected with the donor plasmids and the original non-TCOF1-specific pX330 did not show visible evidence of knock-in via genotyping PCR, and comparison of cells before and after double selection showed that selection resulted in reduction of the wild-type alleles and predominance of the knock-in alleles in the overall pool (Figure 1B).

### DD-TCOF1 expression is regulated by the concentration of Shield-1

To assess whether DD-TCOF1 was expressed in the knock-in cells, we incubated cells with differing concentrations of Shield-1 and collected samples for Western after 24 hours. DD-TCOF1 expression was regulated in a dose-dependent manner, with 1000 nM Shield-1 resulting in over 3-fold more protein than the absence of Shield-1 (Figure 2A). By comparison, TCOF1 expression was not increased by Shield-1 in parental wild-type 293T cells (Figure 2A). We then observed the kinetics of DD-TCOF1 destabilization or stabilization. We either removed Shield-1 from knock-in cells initially in 1000 nM Shield-1, or added 1000 nM Shield-1 to knock-in cells initially without Shield-1. Removal of Shield-1 was facilitated by a brief incubation in conditioned media supplemented with 5 µM FKBP12 (the F36V mutant, from which the DD domain was derived), which acts as a strong thermodynamic sink for residual Shield-1 [21]. Within 24 hours, DD-TCOF1 levels were destabilized or stabilized over 3-fold (Figure 2B and C). The over 3-fold difference we observed was consistent with the 3.7-fold difference in mean fluorescence intensity previously observed for a DD-tagged YFP targeted to the nucleus [25].

### DD-TCOF1 retains the nucleolar localization of wild-type TCOF1

**Figure 3:**
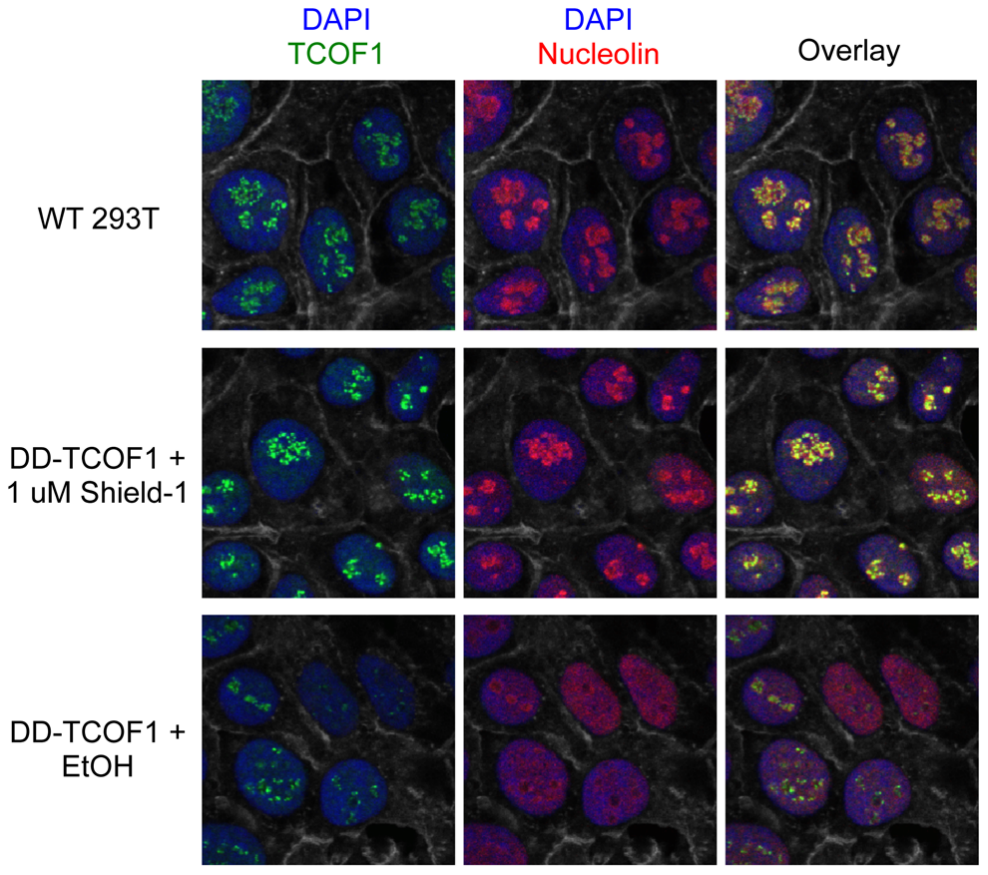
DD-TCOF1 retains the nucleolar localization of wild-type TCOF1. Knock-in cells in Shield-1 or ethanol vehicle alone and parental wild-type 293T cells were stained and imaged as described in Materials and Methods. A single representative z-slice confocal image is shown. The visible grayscale cell boundaries represent F-actin (stained with phalloidin).

**Figure 4:**
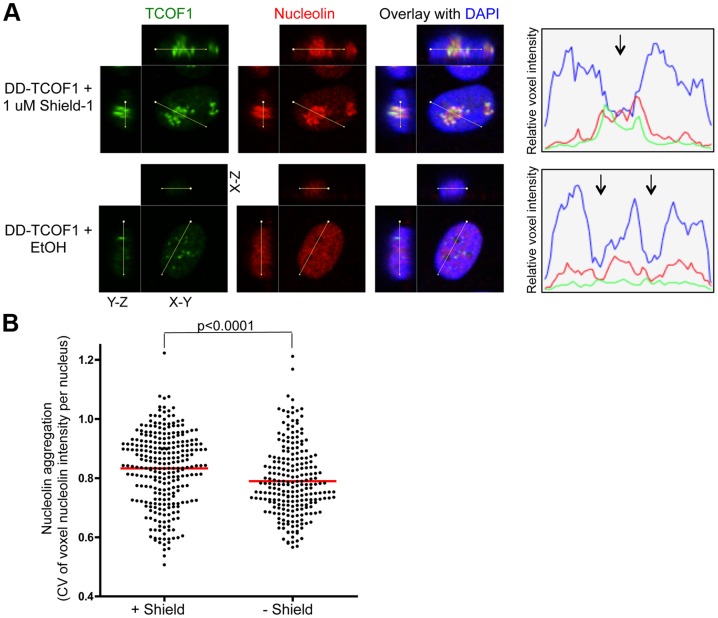
Destabilization of TCOF1 results in dispersal of nucleolin. (**A**) The relative fluorescence intensities across the indicated lines are shown in the graph, and the arrows indicate the position of nucleoli as defined by a hole in the DAPI stain. Representative nuclei from knock-in cells in Shield-1 or ethanol vehicle alone are shown. (**B**) The aggregation of nucleolin was measured by the coefficient of variation (CV, stdev/mean) of nucleolin voxel intensities within DAPI-defined nuclei in 3D reconstructions, as described in Materials and Methods. As examples, this CV metric was 0.95 for the +Shield nucleus and 0.71 for the –Shield nucleus in panel A. The population means are indicated by the red lines. There is significantly less nucleolin aggregation in knock-in cell nuclei in the absence of Shield-1, p<0.0001, 2-tailed Student's t-test, n = 272 for +Shield and n = 230 for –Shield.

We then determined whether the DD-tagged protein retained wild-type characteristics. Although the localization of TCOF1 may be dynamic, it is known to be predominantly nucleolar. The DD-TCOF in knock-in cells was indeed nucleolar, as indicated by strong co-localization with nucleolin in the presence of Shield-1 (Figure 3 and 4A). As a further confirmation of the specificity of our anti-TCOF1 antibody, TCOF1 staining in knock-in cells in the absence of Shield-1 was significantly reduced (Figure 3, also p<0.0001 by 2-tailed Student's t-test for +Shield nuclei (n = 272) vs. –Shield nuclei (n = 230) as described in Materials and Methods, data not shown). Unexpectedly, we noticed that upon DD-TCOF1 destabilization, nucleolin itself appeared disaggregated and dispersed throughout the nucleoplasm (Figure 3 and 4A). To quantitatively evaluate this observation, we reasoned that within a given nucleus, while aggregation would lead to polarization of the distribution of voxel intensities and thus increased spread of intensity values, dispersion throughout the nucleoplasm should result in a voxel intensity distribution more evenly clustered around the mean and thus reduced spread of intensity values (e.g., see the vertical spreads of nucleolin voxel intensities in Figure 4A). We therefore used the coefficient of variation (the standard deviation normalized to the mean) of nucleolin voxel intensities within 3D reconstructions of DAPI-defined nuclei as a metric for nucleolin aggregation. Indeed, destabilization of DD-TCOF1 appears to result in reduced nucleolin aggregation (Figure 4B), reminiscent of previous findings that TCOF1 knockdown results in dispersion of Pol I and UBF away from nucleoli [11]. Since it is possible that a mutation specific to clone 1 was able to modulate the functional consequence of TCOF1 destabilization, especially in light of possible off-target effects of the CRISPR/Cas9 system [26]–[31], we confirmed that independent clone 2 also showed destabilization of TCOF1 and significantly reduced nucleolin aggregation (p<0.0001) in the absence of Shield-1 (Figure S4).

### Destabilization of DD-TCOF1 impairs cell growth

**Figure 5:**
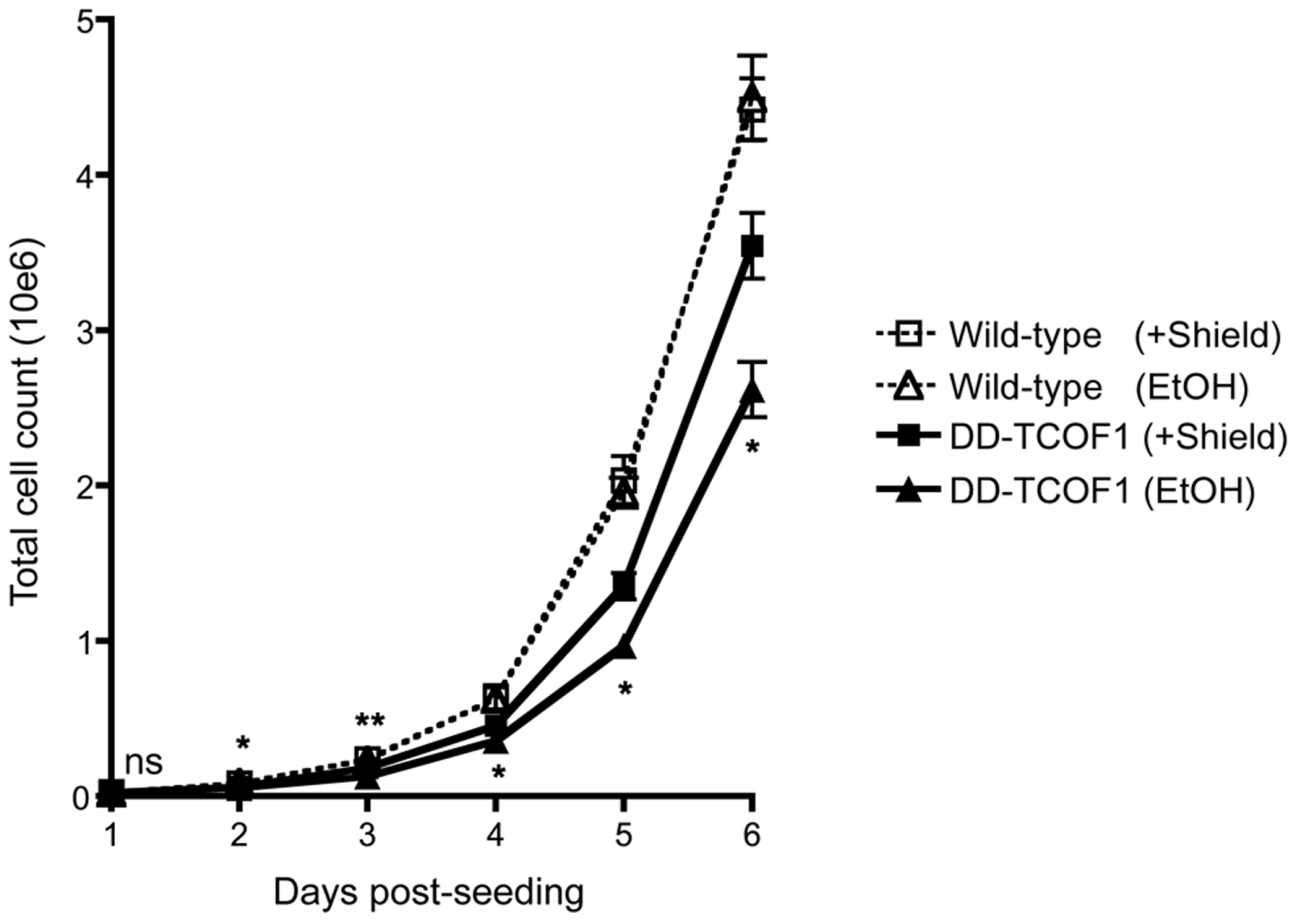
Destabilization of TCOF1 impairs cell growth. The growth of knock-in cells in the presence or absence of Shield-1 was compared as described in Materials and Methods. EtOH represents the vehicle control. Error bars represent SD of 3 replicate samples. The knock-in cell count in the absence of Shield-1 was significantly less than in the presence of Shield-1 except on the first day post-seeding: ns, not significant; *, p<0.05; **, p<0.01, 2-tailed Student's t-test at each time point. The growth of knock-in cells in the presence or absence of Shield-1 was compared two other times with similar results.

Since TCOF1 is an essential gene important for ribosome biogenesis among other functions, we expected that destabilization of DD-TCOF1 should impair cell growth, although immortalized cell lines might be in general more resistant to the effects of TCOF1 gene disruption. Indeed, we found that while the presence or absence of Shield-1 did not affect the growth of parental wild-type 293Ts, the absence of Shield-1 (which leads to degradation of TCOF1) resulted in significantly reduced growth for the knock-in cells (Figure 5).

## Discussion

The CRISPR/Cas9 homing endonuclease system and its utility for efficient genomic engineering has rapidly instigated widespread exploration for various applications. Here we report that the high efficiency of homologous recombination mediated by CRISPR/Cas9 allowed rapid and complete knock-in of destabilization domain-tagged TCOF1 in the hyperploid human 239T cell line. Expression of DD-TCOF1, which retained the subcellular localization and at least the growth-related function of the untagged protein, could be regulated by the stabilizing compound Shield-1. This knock-in strategy likely represents a generalizable and reasonable strategy in other diploid and hyperploid mammalian cells, particularly for essential genes that cannot be knocked out without severely affecting cell viability.

Although siRNA-mediated knockdown of TCOF1 has been attempted in the past, protein reduction is significantly apparent after four days [10], [11]; the long time required to achieve significant knockdown may allow secondary effects of reduced viability to accumulate, which might confound the interpretation of results. Our present work demonstrates that efficient complete knock-in of DD-tagged TCOF1 via the CRIPSR/Cas9 system enables relatively rapid knockdown of protein levels upon removal of the stabilizing Shield-1 compound, or conversely, rapid upregulation of TCOF1 levels upon addition of Shield-1. In principle, the use of siRNA (or other means to regulate mRNA expression such as a regulatable Tet system) in knock-in cells in combination with Shield-1 removal might result in an even more dramatic decrease in DD-TCOF1 protein levels, since the basal level of DD-TCOF1 is due to the continual production of more protein from the mRNA. By reducing the mRNA itself, siRNA may enhance the decrease in DD-TCOF1 levels upon Shield-1 removal.

Importantly, although random donor insertion or off-target mutations were possible, biological interrogation of TCOF1 function could occur in an isogenic cellular background except for the modulation of TCOF1 levels via Shield-1. An independent clone confirmed our findings (Figure S4). Although we used the wild-type Cas9 endonuclease that catalyzes both nicks and double-strand breaks (DSBs), the use of a mutant Cas9 that only nicks may be used to avoid DSB-mediated non-homologous end joining mutations in off-target sites [26], [30], [32].

Although we used the endogenous promoter to drive expression of DD-TCOF1 in this study, a constitutive strong or weak promoter could also be added to the donor and used, depending on the context, in order to modify the dynamic range of DD-tagged protein, from the basal level to the maximally stabilized level of expression at reasonable Shield-1 concentrations. A stronger promoter would likely shift the entire dynamic range higher, while a weaker promoter would shift the dynamic range lower. Such a design would also allow the use of separate promoters for the antibiotic resistance gene and the DD-tagged gene of interest, thus facilitating the removal of the antibiotic gene via Cre/LoxP or FLP/FRT recombination if desired.

One requirement of the FKBP-based destabilization domain for our knock-in strategy for essential genes is that Shield-1 must be maintained on the knock-in cells to maintain cell growth and viability, unless the basal level of DD-tagged protein is sufficient to maintain growth (as in fact appears to be the case for our TCOF1 knock-in clone); this may become cost-prohibitive as Shield-1 is relatively expensive. We also acknowledge the possibility that we may have selected for clones that can tolerate constitutive reduction in TCOF1 protein levels. Fortunately, similar effective DD tag systems may be more cost-effective, such as an *E. coli* dihydrofolate reductase (ecDHFR)-based destabilization domain that is stabilized by the relatively inexpensive trimethoprim [33]. The ecDHFR-based and other recent systems are also orthogonal to the FKBP-based system [33], [34], which would allow independent modulation of more than one protein of interest. Systems that allow conditional degradation rather than stabilization in the presence of compound represent another promising approach [35].

## Supporting Information

Figure S1 **Position of Cas9 guide RNA sequence and genotyping primers in the human genomic sequence.**
(PDF)

Figure S2 **Annotated sequence of knock-in donor constructs.**
(PDF)

Figure S3 **Homologous recombination in both 5′ and 3′ homology arms.** Genotyping PCR was performed with the same forward primer shown in Figure 1B, but with a reverse primer beyond the 3′ homology arm.
(TIF)

Figure S4 **Confirmation of dispersal of nucleolin with an independent clone.** Destabilization of DD-TCOF1 in clone 2 was confirmed by Western analysis of cells incubated in either ethanol vehicle control or 1 µM Shield-1. The analysis of nucleolin dispersal was performed as described for Figure 4. CV, coefficient of variation (stdev/mean); p<0.0001, 2-tailed Student's t-test, n = 338 for +Shield and n = 243 for –Shield.
(TIF)
