# Supplementary material for: CRISPR/Cas9 Allows Efficient and Complete Knock-In of a Destabilization Domain-Tagged Essential Protein in a Human Cell Line, Allowing Rapid Knockdown of Protein Function
Source: PLoS One. 2014 Apr 17;9(4):e95101. doi: 10.1371/journal.pone.0095101 (PMC3990584; doi:10.1371/journal.pone.0095101)
Supplement: Figure S1 — Position of Cas9 guide RNA sequence and genotyping primers in the human genomic sequence. (PDF) [file pone.0095101.s001.pdf]

The human genomic sequence flanking TCOF1 exon 1 is shown.

Exon 1
TCOF1 start codon
Genotyping primers
Guide RNA sequence (inserted into pX330)

…CCCTCATTTGTTCCCTCTTCCCCACCTCACCCCACCCCACCTGGACAGCCCAGCAGCG
CAGACTAGGGGTTCTGGGTTTAGGGTTCCCAGGCAATAGCAGGGTGTTTTCTCATGCCT
AGACTCATGCAGTGCCCTCTGCCAGTCCTCTCCTTTGCACCCAGCAATTTCGGGTGTCT
TTATGCCTCAGCTTAGATATCTCTCTGGGGTTCGGTGCTCCTCCTTGGGGCGCCCATAG
GCTGCATCTGTCCCTGACAAGGCACACAAGATATTGAACTATAGTTGCTTGGTTACGGA
CAGTCGTCTCTGTGAGCTTTGAGGGCAACTGCCATGTATTATGCATCTTTTGAATCCCC
CTAGCCTAGCCGGGTGCTAGCCCAAGTTAGGTGCTTATTACAGATTTGTTCAGTTAGCG
AATGAATCAATGAATAAACTGGCAATTCGTGTCTGGTTCATGGGCACCAAAAACCGTGC
GAAATGGATAAATGAGCAGGTAATGGCAGTACAAAGTTCGGATCTAGAGAAAGATCCAG
ATTAGGGAGTTCAGAGATGAGTAAAACGCAGACCCAGCCATCAGAGAATTAACAAGGAA
GATAAATCTCTTCAAGAAGCCAGCCGGAAGGATTAAAAGAGAGTCGTCCAAGTCCCGGC
CGGCCCCGGGGGCGGGTCCCTGTCTAGCCCCGCATGCTCCGCGCCAATGGGCGGTATTG
TTGATCACGAGTCTCCGCCCCCTTCGCCTTGAGGGGCGGGGCCGGCCACTCCCGGAGAG
GGGACTACGTTTCCCGGCGCGCCGCGCGGCCGGAGC**GAAAGAGGAGCCGGAAGTGGG**
GCGCGCGAGGTCTAAGGGCGCGAGGGAAGTGGCGGGCGGGGACTAAGGCGGG
GCGTGCAGGTAGCCGGCCGGCCGGGGGTCGCGGTATGGCCGAGGCCAGGAA
GCGGCGGGAGCTACTTCCCCTGATCTACCACCATCTGCTGCGGGCTGGCTAT
GTGCGTGCGGCGCGGGAAGTGAAGGAGCAGAGCGGCCAGGTAAGCGTTCGTGGG
CCGTGTGCGAGGGCCGCGTGCAAGATGTGGAGATCAGCGGCCCGCGGCCCGCGCCCCGT
CCCCAGGCGACCCGGCAGGCGCCCGGAGCCGGGTCCCGCAGTGCTCGACGGCGCGGCCA
GGGGTACCGGAGGAGCCGCAATCTCTGCCTTCCCACTGCGACTTCAGTTCCCCTGGGCC
TCACTTTCCTCATCCGGGCAGTGGGTTGGGCCAGAAAGTGTGCTCGCAGGGGCCGAACT
TTGTAATTCCCGCTTCCCTTACCTCCACGCCCTTCCTGGTAGCGGGTATTTTAAGTTTC
CTAAGTCTCCCGCCACGTGGCTAGGCTCTGCGCGGCCCCCCCTGGGGCAAGGAGGTTGC
TGCGAGTCTCGGGGGTGGATTGCGACCAGCCCCTCGGGGGAGCGCCTGGAAAAGGGACC
CTACAGCCTCTTAGTTGAGCTGGAACTCAGGCCTTTAGCGATTGTCTGTACTGAGACGG
TCAGCTTTGGTACTCGAACTCGTGGTACAGTCATTTCTTACTTGATTAATTCAACTGTT
AATCCGGTACTCAGGGTGCTTCCTGGTGTTCGAATGGTGAGCACCATCTCTCAGCCCTC
CTGGAGCTTACACTCTAGTAAGGTAGACAAATCAAAGAATCACAAAAGTGACTATATGC
TTAGCACCGCAATAAGTGTTAAGAATGAGAGGGATGGGCCGGGCACGGTAGCTCACGCC
TGTAATCCCAGCACTTAGGGAGGCCGAGACTAGCGGATCACCTGAGGTCAGAAGTTCGA
GACCAGCCTGGCCAACATGGTGAAACACCGTTTCTACTAAAAATACAAAAATTAGCTGG
GCATGTTGGCGCACGCCTGTAATCCTGGCTACTCATGTGGCTGAGGCACAAGAATTGCT
TGAACCCGGGAGGCAGAGGTTGCAATGAGTCAAGATCGTGCCACTGCACTCCAGCCTGG
GCGATAAAGCGAGACTCTGCCTCAAAAAAAAAAGAGAGATGGATGGCAGATTGCCCTGG
TCAGGAATAGCTGAGGTGTAAATGCCTTGTGGTGGGAGGGAACTTGTGTACACACTGGC
CTGGAGAAAGTCCACTCTCTTGAGCCCAGGAATTTGAGGATACAGTATGAGCCATGATC
ATGCCACTGCACTCCAGCCTGGGCAACAGAGCAAGACACTGTCTCAAAAAAAAATTTTT
TTTAAGTCCATAGCAGGCCAGGTACGGTAGCTCACGCCTGTAATCCCAGCACTTTGGGA
GGCCGAGGCAGGCGGATCACTTGAGGCCAG…
