# Supplementary material for: CRISPR/Cas9 Allows Efficient and Complete Knock-In of a Destabilization Domain-Tagged Essential Protein in a Human Cell Line, Allowing Rapid Knockdown of Protein Function
Source: PLoS One. 2014 Apr 17;9(4):e95101. doi: 10.1371/journal.pone.0095101 (PMC3990584; doi:10.1371/journal.pone.0095101)
Supplement: Figure S2 — Annotated sequence of knock-in donor constructs. (PDF) [file pone.0095101.s002.pdf]

The following donor insert was inserted into the PCR2.1 vector (Invitrogen).

BamHI and EcoRI restriction sites

Homology arms (all underlined sequence, regardless of color)

TCOF1 exon 1

Puromycin resistance (alternative Blast^R is below)

GSG linker

P2A ribosomal skipping sequence

Destabilization domain

3XGGGGS linker

TCOF1 start codon

GGATCC
CAGCAATTTCGGGTGTCTTTATGCCTCAGCTTAGATATCTCTCTGGGGTTCGGTGCTCC
TCCTTGGGGCGCCCATAGGCTGCATCTGTCCCTGACAAGGCACACAAGATATTGAACTA
TAGTTGCTTGGTTACGGACAGTCGTCTCTGTGAGCTTTGAGGGCAACTGCCATGTATTA
TGCATCTTTTGAATCCCCCTAGCCTAGCCGGGTGCTAGCCCAAGTTAGGTGCTTATTAC
AGATTTGTTCAGTTAGCGAATGAATCAATGAATAAACTGGCAATTCGTGTCTGGTTCAT
GGGCACCAAAAACCGTGCGAAATGGATAAATGAGCAGGTAATGGCAGTACAAAGTTCGG
ATCTAGAGAAAGATCCAGATTAGGGAGTTCAGAGATGAGTAAAACGCAGACCCAGCCAT
CAGAGAATTAACAAGGAAGATAAATCTCTTCAAGAAGCCAGCCGGAAGGATTAAAAGAG
AGTCGTCCAAGTCCCGGCCGGCCCCGGGGGCGGGTCCCTGTCTAGCCCCGCATGCTCCG
CGCCAATGGGCGGTATTGTTGATCACGAGTCTCCGCCCCCTTCGCCTTGAGGGGCGGGG
CCGGCCACTCCCGGAGAGGGGACTACGTTTCCCGGCGCGCCGCGCGGCCGGAGC**GAAAG**
AGGAGCCGGAAGTGGGGCGCGCGAGGTCTAAGGGCGCGAGGGAAGTGGCGGGCGGGGAC
TAAGGCGGGGCGTGCAGGTAGCCGGCCGGCCGGGGGTCGCGGGT
ATGACCGAGTACAAGCCCACGGTGCGCCTCGCCACCCGCGACGACGTCCCCAGGGCCGT
ACGCACCCTCGCCGCCGCGTTCGCCGACTACCCCGCCACGCGCCACACCGTCGATCCGG
ACCGCCACATCGAGCGGGTCACCGAGCTGCAAGAACTCTTCCTCACGCGCGTCGGGCTC
GACATCGGCAAGGTGTGGGTCGCGGACGACGGCGCCGCGGTGGCGGTCTGGACCACGCC
GGAGAGCGTCGAAGCGGGGGCGGTGTTCGCCGAGATCGGCCCGCGCATGGCCGAGTTGA
GCGGTTCCCGGCTGGCCGCGCAGCAACAGATGGAAGGCCTCCTGGCGCCGCACCGGCCC
AAGGAGCCCGCGTGGTTCCTGGCCACCGTCGGCGTCTCGCCCGACCACCAGGGCAAGGG
TCTGGGCAGCGCCGTCGTGCTCCCCGGAGTGGAGGCGGCCGAGCGCGCCGGGGTGCCCG
CCTTCCTGGAGACCTCCGCGCCCCGCAACCTCCCCTTCTACGAGCGGCTCGGCTTCACC
GTCACCGCCGACGTCGAGGTGCCCGAAGGACCGCGCACCTGGTGCATGACCCGCAAGCC
CGGTGCC
GGATCCGGA
GCCACGAACTTCTCTCTGTTAAAGCAAGCAGGAGACGTGGAAGAAAACCCCGGTCCC
ATGGGAGTGCAGGTGGAAACCATCTCCCCAGGAGACGGGCGCACCTTCCCCAAGCGCGG
CCAGACCTGTGTGGTGCACTACACCGGGATGCTTGAAGATGGAAAGAAAGTCGATTCCT
CCCGGGACAGAAACAAGCCCTTTAAGTTTATGCTAGGCAAGCAGGAGGTGATCCGAGGC
TGGGAAGAAGGGGTTGCCCAGATGAGTGTGGGTCAGAGAGCCAAACTGACTATATCTCC
AGATTATGCCTATGGTGCCACTGGGCACCCAGGCATCATCCCACCACATGCCACTCTCG
TCTTCGATGTGGAGCTTCTAAAACCGGAA

GGT GGA GGT GGA TCT GGT GGA GGT GGA TCT GGT GGC GGC GGT TCA
ATGGCCGAGGCCAGGAAGCGGCGGGAGCTACTTCCCCTGATCTACCACCATCTGCTGCG
GGCTGGCTATGTGCGTGCGGCGCGGGAAGTGAAGGAGCAGAGCGGCCAGGTAAGCGTTC
GTGGGCCGTGTGCGAGGGCCGCGTGCAAGATGTGGAGATCAGCGGCCCGCGGCCCGCGC
CCCGTCCCCAGGCGACCCGGCAGGCGCCCGGAGCCGGGTCCCGCAGTGCTCGACGGCGC
GGCCAGGGGTACCGGAGGAGCCGCAATCTCTGCCTTCCCACTGCGACTTCAGTTCCCCT
GGGCCTCACTTTCCTCATCCGGGCAGTGGGTTGGGCCAGAAAGTGTGCTCGCAGGGGCC
GAACTTTGTAATTCCCGCTTCCCTTACCTCCACGCCCTTCCTGGTAGCGGGTATTTTAA
GTTTCCTAAGTCTCCCGCCACGTGGCTAGGCTCTGCGCGGCCCCCCCTGGGGCAAGGAG
GTTGCTGCGAGTCTCGGGGGTGGATTGCGACCAGCCCCTCGGGGGAGCGCCTGGAAAAG
GGACCCTACAGCCTCTTAGTTGAGCTGGAACTCAGGCCTTTAGCGATTGTCTGTACTGA
GACGGTCAGCTTTGGTACTCGAACTCGTGGTACAGTCATTTCTTACTTGATTAATTCAA
CTGTTAATCCGGTACTCAGGGTGCTTCCTGGTGTTCGAATGGTGAGCACCATCTCTCAG
CCCTCCTGGAGCTTACACTCTAGTAAGGTAGACAAATCAAAGAATCACAAAAGTGACTA
TATGCTTAGCACCGCAATAAGGGCCGGCCAAAAGGGC
GAATTC

Blasticidin resistance:
ATGGCCAAGCCTTTGTCTCAAGAAGAATCCACCCTCATTGAAAGAGCAACGGCTACAAT
CAACAGCATCCCCATCTCTGAAGACTACAGCGTCGCCAGCGCAGCTCTCTCTAGCGACG
GCCGCATCTTCACTGGTGTCAATGTATATCATTTTACTGGGGACCTTGTGCAGAACTC
GTGGTGCTGGGCACTGCTGCTGCTGCGGCAGCTGGCAACCTGACTTGTATCGTCGCGAT
CGGAAATGAGAACAGGGGCATCTTGAGCCCCTGCGGACGGTGCCGACAGGTGCTTCTCG
ATCTGCATCCTGGGATCAAAGCCATAGTGAAGGACAGTGATGGACAGCCGACGGCAGTT
GGGATTCGTGAATTGCTGCCCTCTGGTTATGTGTGGAGGGC
