# Supplementary figures and images for: CRISPR/Cas9 Allows Efficient and Complete Knock-In of a Destabilization Domain-Tagged Essential Protein in a Human Cell Line, Allowing Rapid Knockdown of Protein Function
Source: PLoS One. 2014 Apr 17;9(4):e95101. doi: 10.1371/journal.pone.0095101 (PMC3990584; doi:10.1371/journal.pone.0095101)

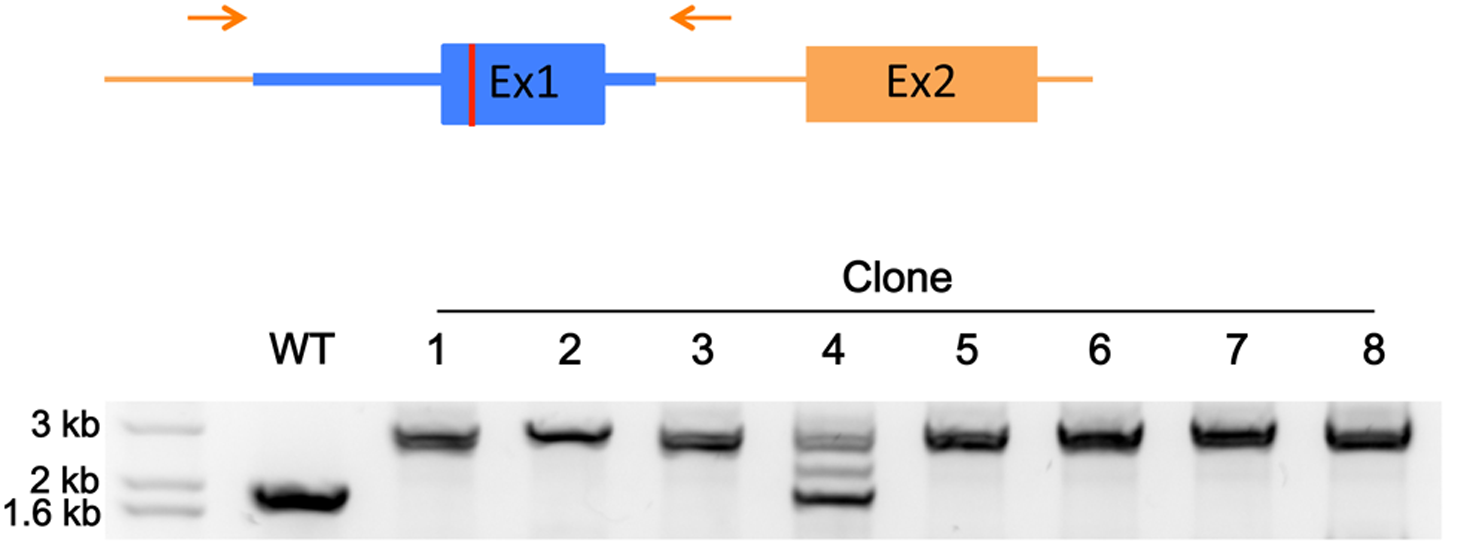

Supplement: Figure S3 — Homologous recombination in both 5′ and 3′ homology arms. Genotyping PCR was performed with the same forward primer shown in Figure 1B, but with a reverse primer beyond the 3′ homology arm. (TIF) [file pone.0095101.s003.tif]

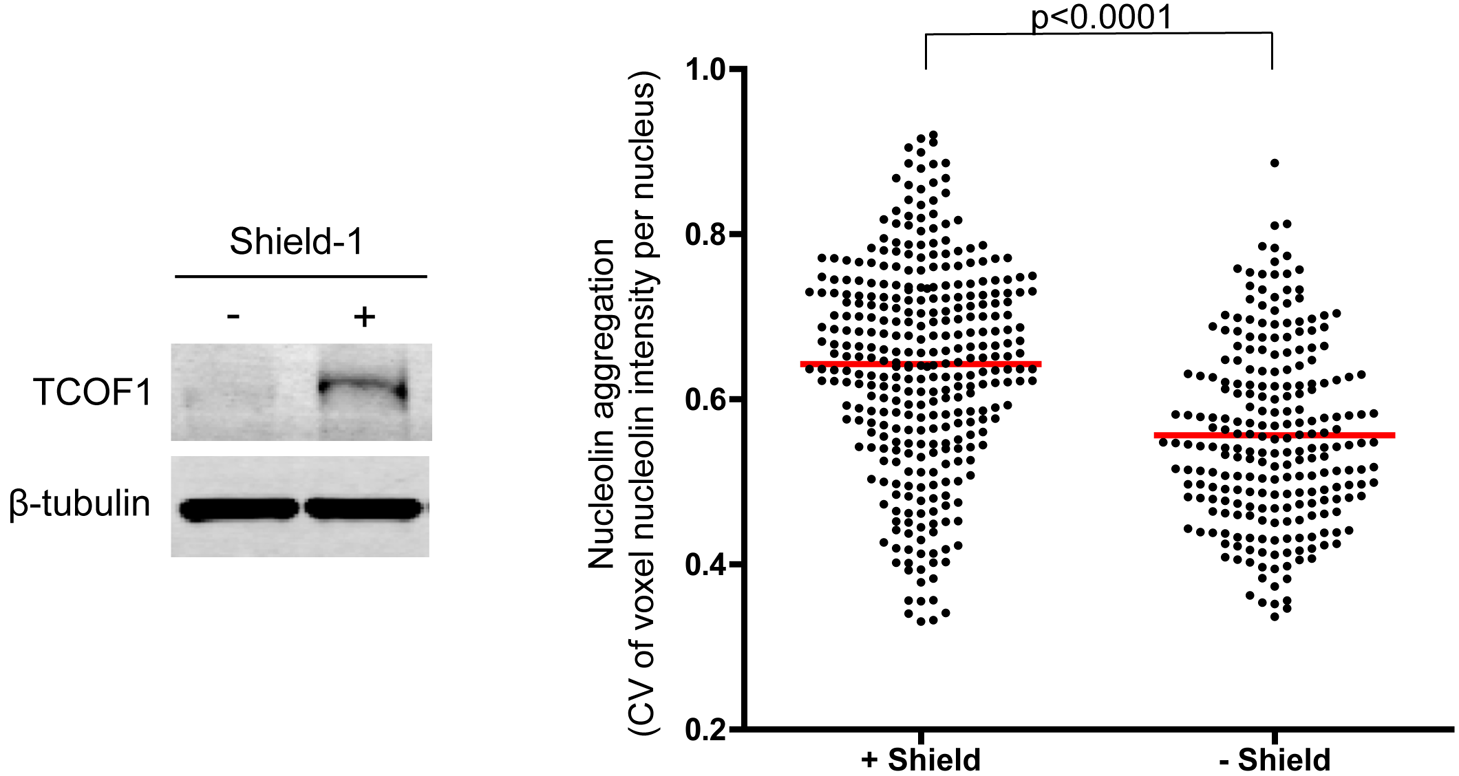

Supplement: Figure S4 — Confirmation of dispersal of nucleolin with an independent clone. Destabilization of DD-TCOF1 in clone 2 was confirmed by Western analysis of cells incubated in either ethanol vehicle control or 1 µM Shield-1. The analysis of nucleolin dispersal was performed as described for Figure 4. CV, coefficient of variation (stdev/mean); p<0.0001, 2-tailed Student's t-test, n = 338 for +Shield and n = 243 for –Shield. (TIF) [file pone.0095101.s004.tif]
